# Assessing Nutritional Status and Frailty among Poor Elderly Individuals in Requena del Tapiche, Peru

**DOI:** 10.3390/nu15173840

**Published:** 2023-09-02

**Authors:** María Teresa Murillo-Llorente, Blanca Lafuente-Sarabia, Jennifer Samper de la Paz, Merita Flores-Púa, Manuel Tejeda-Adell, María Ester Legidos-García, Marcelino Perez-Bermejo

**Affiliations:** 1 SONEV Research Group, School of Medicine and Health Sciences, Catholic University of Valencia San Vicente Mártir, C/Quevedo nº 2, 46001 Valencia, Spain; mt.murillo@ucv.es (M.T.M.-L.); ester.legidos@ucv.es (M.E.L.-G.); 2 School of Medicine and Health Sciences, Catholic University of Valencia San Vicente Mártir, C/Quevedo nº 2, 46001 Valencia, Spain; blanca.lafuente@mail.ucv.es (B.L.-S.); jennifer.samper@ucv.es (J.S.d.l.P.); manuel.tejeda@ucv.es (M.T.-A.); 3 Nurse Coordinator of the “Padre Nicolás Giner” Health Cente, Requena del Tapiche 16341, Peru; luzeva923@hotmail.com

**Keywords:** elderly, malnutrition, mobility limitation, frail elderly, dependency, Peru

## Abstract

Frailty is a biological syndrome that leads to a loss of physiological reserve, increasing susceptibility to adverse health events. In the Peruvian Amazon, the elderly live with hardly any economic resources, presenting a caloric deficit that is related to functional and cognitive deterioration. Our objective was to identify the health needs of elderly people living in extreme poverty in Requena (Peru) by means of a geriatric assessment of the nutritional and functional spheres to design, in the future, a cooperation project appropriate to the needs detected. This is an observational, descriptive, and cross-sectional study. Sixty participants were included, and sociodemographic and functional status variables were analyzed using the MNA and Barthel scales and the Get Up and Go test. The mean age of the participants was 79 ± 6.67 (women 55% and men 45%), where 60% had frailty. A statistically significant relationship was found between the MNA scores and Barthel test. Eighty-five percent were malnourished or at risk and thirteen percent had total or moderate dependence. We conclude that the nutritional status of the elderly was deficient. The high degree of living alone in which they live forces them to maintain their independence and their walking stability is normal. The situation of frailty exceeds the national average, a situation that has repercussions for their quality of life. We found a statistically significant association between nutritional status, dependence, and frailty. The better-nourished elderly are less frail and less dependent.

## 1. Introduction

The United Nations General Assembly declared the period of 2021–2030 as the Decade for Healthy Aging [1] and asked the WHO to take the lead in its implementation. It is a 10-year project aimed at promoting longer and healthier lives. It supports the fulfillment of the 2030 Agenda for Sustainable Development and the Sustainable Development Goals.

Old age is characterized by the emergence of several complex health conditions commonly referred to as geriatric syndromes. They are usually the consequence of multiple underlying factors, including, among others, reduced mobility, falls, urinary incontinence, delirious states, and pressure ulcers [2]. The combination of age-associated diseases exposes the elderly to dependency, adverse outcomes, and frailty, which is the inability to endure illness without loss of function [3].

The frail elderly are, therefore, vulnerable individuals because of the cumulative decline of multiple physiological systems over their lifetime [4]. According to Kane et al. [5], frailty can be viewed as a state of physiological decline that increases susceptibility to adverse health outcomes. This loss of physiological reserve means that even small stressors can lead to disability and death in frail individuals.

It is important to differentiate between the frail elderly and those at risk of becoming frail, as the frail elderly are those with advanced age who undergo several changes because of aging in the different organs and systems that confer physiological loss accompanied or not by disease [6,7]. Fried [8] established the criteria for identifying frailty with the presence of unintentional weight loss, muscle weakness, slow gait, and a low level of physical activity.

The Comprehensive Geriatric Assessment (CGA) is an essential tool in the evaluation of the health status of the elderly, which can detect problems that have not been detected until now [9]. It is a multidimensional, interdisciplinary diagnostic process designed to identify and quantify medical problems, and evaluate functional and psychosocial capacities, to reach a global treatment plan, optimize the use of care resources, and guarantee the continuity of long-term care [10].

Elderly people are part of society, although they sometimes experience the effect of loneliness [11]. Age brings the memory of lived experiences and situations of joy or sadness, which is why it is important to provide holistic care for elderly people, not only for their physical well-being, but also for their psychological, emotional, and spiritual well-being [12]. It is important to attend to their needs, as well as to exercise their memory and social skills so that they can enjoy a happy routine and an active life.

The United Nations defines poverty as an income of less than $2 per day and extreme poverty as surviving on less than $1.25 per day [13]. In the Department of Loreto (Peru), where Requena del Tapiche is located, we find 28.3–32.7% of people in poverty and 5.8–7.9% of people in extreme poverty, above the national percentage [14]. In addition, Requena has the lowest average age of mortality of all Peru, with a difference of 15 years with the Departments of Ancash, Lima, and Moquegua, an average mortality of 76 years, and mostly deaths due to communicable diseases (216.9 × 100,000 inhabitants) [14]. These data show the need to enhance the quality of life and autonomy of the elderly, since most of them live alone or have few family ties, as well as to provide support information to caregivers in cases where they live with them. After the last few years of the pandemic, which, in the Loreto region, were devastating, the elderly who survived SARS-CoV-2 suffer from muscular, respiratory, digestive, or mental affections [15].

Therefore, the objective of this study was to carry out a nutritional assessment of the elderly in the program of care for the elderly in extreme poverty of Caritas Requena (Peru). This first step is necessary and very useful for drawing up a list of problems that will later be prioritized to establish a realistic plan of therapeutic intervention and care. The geriatric assessment scales evaluate different spheres of a person. They provide us with a holistic view that guarantees adequate, effective, and quality care [16], which will allow us to identify people at high risk of frailty so that health centers can carry out a more exhaustive follow-up [10].

## 2. Materials and Methods

This is an observational, descriptive, and cross-sectional study. The study population was the entire elderly population of the city of Requena (Peru) included in the program of care for the elderly in extreme poverty carried out by Pastoral Social Caritas Requena.

Caritas is an organization of the Catholic Church that promotes and leads programs and projects of integral human development in the poorest populations of Peru. Caritas Requena belongs to the “Red Selva” (Selva Network) and is financed by public and private funds, as well as donations. Pastoral Social Caritas Requena carries out programs and projects of integral human development for the benefit of the most vulnerable populations. The program to help elderly people in extreme poverty is well known to the population. To include elderly people in the program, they have to apply to the coordinator of the Padre Nicolás Giner Health Center, after which a home visit is made to assess their state of health and living conditions, government aid received, social support, etc. This includes people who have no family help or support, who eat two or fewer meals a day, who do not have a mattress or mat to sleep on, who do not receive or take vitamin supplements, who, despite their advanced age, need to work to survive, and whose income is less than 200 soles a month (about 54 USD).

The sample was chosen by convenience without sampling so that all people over 65 years of age in the Caritas program were included in the study. The nurse coordinator of the Padre Nicolás Giner Health Center planned the daily visits to the homes of the elderly by geographical areas, where the scales were passed and the necessary data were taken. In addition, these visits allowed direct observation of the conditions of the homes. Every day, the working group visited between 4 and 5 homes, assessing the nutritional status and frailty of the elderly living in them, as well as detecting risk factors. The visits took place in privacy.

The direct data collection period was between 28 June and 15 July 2022. The study was conducted using the PAPI (Paper and Pen Personal Interview) direct survey method. The data were collected directly by a group of nurse volunteers belonging to the Catholic University of Valencia (UCV) and the nurse coordinator of the health center that Caritas has in Requena. Since the end of 2019, joint projects have been developed between Caritas Requena and UCV.

### 2.1. Ethical Approval and Consent to Participate

The project was approved by the Research Ethics Committee of the Catholic University of Valencia (approval code UCV/2021-2022/202), and written informed consent was obtained from all participants. This study complies with the principles established in the Declaration of Helsinki.

### 2.2. Variables and Rating Scales

Sociodemographic variables (age, sex, and district of residence) and clinical variables (family history, diagnosed diseases, allergies, and hospitalizations) were obtained. In addition, the following rating scales were used:

#### 2.2.1. Frailty Evaluation Using the Fried Assessment

Fried proposed the classification of an individual as frail through the presence of 3 or more of the 5 phenotype criteria: slowed gait speed, poor physical activity, self-reported physical fatigue, unintentional weight loss, and muscle weakness (estimated by prehension strength) [8].

#### 2.2.2. Mini Nutritional Assessment Scale

The aging of the population is one of the factors influencing the increase in the prevalence of malnutrition, as the elderly are a group at risk due to their biological, psychological, and social characteristics. Malnutrition is underdiagnosed in geriatrics, and this was the origin of the development of the Mini Nutritional Assessment (MNA) scale [17,18]. It is a structured and validated nutritional assessment method for the population at the hospital, residential, or community level dedicated to obtaining an assessment of the nutritional status among malnourished elderly people or those at risk of malnutrition [19,20].

The test consists of 18 questions divided into four nutritional areas that perform a global assessment through questions related to lifestyle, medication, physical and mental status, dietary history through questions related to daily dietary intake and intake problems, and a self-perception of health, together with an anthropometric assessment (BMI, brachial, and thigh circumference), as well as weight loss [5,6,21,22,23,24]. It consists of two parts. A screening test and a post-screening test are necessary to obtain an accurate assessment of the patient’s nutritional status [25]. Its elaboration yields a final numerical quantity. If this number is greater than or equal to 24, the elderly person has a satisfactory nutritional status. If it is between 17 and 23.5, the patient is at risk of malnutrition, and if it Is less than 17, the elderly person has a poor nutritional status.

#### 2.2.3. Barthel Index

The Barthel index is a scale that assesses a patient’s level of independence [26], through their ability to perform Basic Activities of Daily Living (BADL). This scale can be performed in several ways: self-administered, by direct observation, or by direct questioning of the person or caregiver. The basic activities of daily living that it assesses are eating, washing, dressing, bowel movements, urination, toilet use, transferring, ambulation, and climbing/descending stairs.

This scale is very useful thanks to its validity, sensitivity, and reliability for describing the functional status of the elderly [26,27,28,29] and is considered the most useful scale for assessing ADLs due to its characteristics, low cost, and potential usefulness for monitoring the evolution of elderly patients [30,31,32,33]. It is scored from 0 to 100 and defines 4 categories of dependence: total dependence, less than 20; severe, between 20 and 40; moderate, from 40 to 55; and mild, greater than 60.

#### 2.2.4. Timed Get up and Go Test

This test assesses the risk of falling and is performed by timing the time it takes the patient to get up from a chair with armrests, walk 3 m, and sit back down in their chair. It is interpreted as normal if it takes less than 10 s, normal for elderly or frail elderly if it takes between 11 and 20 s, needs help if it takes more than 20 s, and high risk of falls if it exceeds 30 s [34].

### 2.3. Statistical Analysis

Discrete variables are presented as absolute values and percentages. Continuous variables are expressed as means and standard deviations. A comparison between categorical variables was performed using the Chi^2^ test. To predictively estimate the relationships between the different variables and frailty, analyses based on the logistic regression model were performed. The results are presented as the odds ratio (OR). The Mann–Whitney test was performed to find differences between two quantitative variables and Spearman’s coefficient to correlate them. The Kruskal–Wallis test was used to find differences between three or more groups. For all tests, a significance level of less than 0.05 was accepted in bilateral contrast. Data analysis was performed using SPSS v.23 software (SPSS Inc., Chicago, IL, USA).

## 3. Results

The final sample analyzed was 60 elderly people in extreme poverty. A total of 27 were male (45%) and 33 were female (55%), reflecting parity among participants (z test; *p* = 0.273). The mean age was 79.2 years (SD = 6.67), with a range between 60 and 100 years. Ninety percent of the participants were between 70 and 90 years of age. Table 1 describes the sociodemographic characteristics of the sample.

Three participants reported three types of allergies: to cassava, to ají (a type of hot bell pepper), or to the sun. The rest did not know if they had any type of allergy, including drug allergies. The family history observed included lung disease, pneumonia, meningitis, digestive diseases, diabetes, cancer, cirrhosis, and cataracts. The causes of hospitalization in the last five years were vomiting, constipation, gall bladder surgery, ear surgery, abdominal hernia, femur fracture, or cardiovascular accident. The vast majority said that they had not undergone surgery. It should be taken into account that, in Requena del Tapiche, there is a tertiary hospital without a surgical area or surgeons, and many cannot afford to travel to the city; thus, they undergo very few surgical procedures.

**Figure 1:**
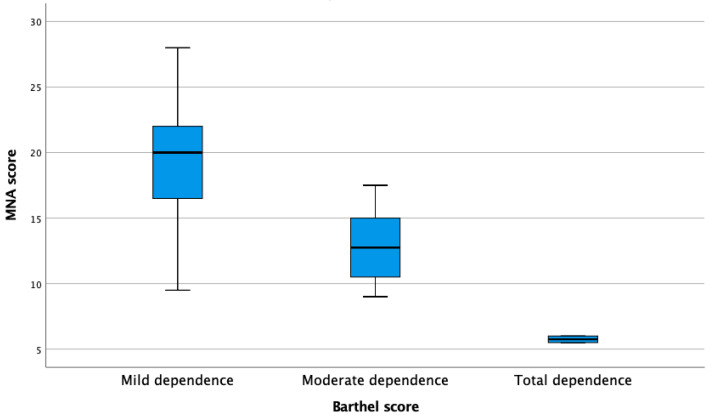
Box-and-whisker plot for dependency of elderly participants in association with their degree of nutritional status according to the MNA.

Table 2 below shows the results of the geriatric assessment scales:

Our binary logistic regression model including frailty as the dependent variable and the MNA, Barthel, and timed get up and go tests as independent variables showed that both MNA (OR = 0.864; *p* = 0.044) and Barthel index (OR = 0.177; *p* = 0.033) were predictor variables of frailty. Table 3 shows the difference in scores according to frailty of the elderly:

The results of the MNA and Barthel tests were positively correlated with each other (Rho = 0.695; *p* < 0.000). Figure 1 shows how the degree of dependence was totally related to nutritional status (Kruskal–Wallis test; *p* < 0.000).

## 4. Discussion

In this paper, we analyzed the dependency and frailty of the elderly in extreme poverty in Requena del Tapiche, Peru. Our results show that most of the elderly had a poor nutritional status, and that, although their degree of dependency was mostly mild, their degree of frailty exceeded the national average in Peru, having a negative impact on their quality of life and being a predictor of the development of diseases.

The nutritional status of the elderly is a widely validated indicator for predicting longevity and quality of life. Rosero and Rosas [35] consider it essential to assess the risk of malnutrition in those over 60 years of age using the MNA scale. In Requena, there are no social–health centers, and all participants lived at home, where 85% were malnourished or at risk of malnutrition. These data reflect the harsh reality of the elderly in Requena, where hunger suffered by the elderly is a totally normalized situation. Similar results were obtained by Deossa-Restrepo and colleagues [36] in their work in Colombia, where 60% were malnourished or at risk. Similarly, Guiraldo et al. [37] found 6.5% malnutrition in the elderly population and 60.1% at risk; this last study is of great interest since, like ours, they studied the state of malnutrition under low socioeconomic conditions. On the other hand, Pereira et al. [38] analyzed the elderly population of the city of Salvador in Brazil, where they found that 66.3% were undernourished. In addition, they observed that the prevalence of this malnutrition was higher in men, a result similar to that obtained in our study.

The degree of dependence of the elderly in Requena was low; we observed that 87% were mildly dependent. These results are similar to those of Diaz [39], where 91% of Ecuadorian participants had the same degree of dependence. Cano-Gutierrez et al. [40] assessed the functional status of elderly people in Bogota, concluding that their cohort had fairly preserved functionality. In our study, we observed a good functional status of the participants directly related to the situation of loneliness they suffered, which forced them to remain functionally active. However, in elderly Spaniards [41], we observed a greater dependence after the age of 60 years (23.4%), a figure that increased to 50% after the age of 85 years. These data may explain the relationship between the cessation of working life and the development of dependence. However, in Requena, they were forced to work until advanced ages, which favored their functional independence.

Most of the participants were in a fragile situation. Analyzing gait stability, we found that they exhibited normal gait quality. Again, the Spanish elderly [42] showed worse results in this area, where the mean number of seconds was 32.2, reflecting a high risk of falls. Mora [43] conducted a study in Colombia with more than 200 independent women with good cognitive capacity, in which he detected that more than 20% had a high risk of falls. However, none of the elderly interviewed in Requena presented such a risk. Our data and those obtained in other studies of elderly Chileans [44,45] reflect a great similarity, since all the results were found to be within normal ranges in the different age and disability ranges. In Requena, only 3% of those interviewed needed assistance in walking. The arrival of old age entails physiological changes in all the systems of a person that affect functionality and, therefore, quality of life. Regarding its prevention, it has been shown that the first protective factor would be to maintain high levels of physical activity (even once the pathological gait has been detected) and to perform adapted sports that would allow for greater control of the pathology, avoiding more rapid development [46]. The performance of sport in Requena is substituted by the work that the elderly perform daily, since it requires very important physical effort, which would explain the high quality of the gait of the participants.

Our results regarding the frailty of the interviewed elderly (60%) are slightly higher than those observed in the study by Pegorari et al. [47], where 55.4% of the Brazilian elderly presented prefrailty. The work of Troncoso-Pantoja and collaborators developed in Chile [48] supports the previous results, since, once again, 59% of the elderly in the sample were pre-fragile and 10.9% were frail. This study highlights the significant correlation between increasing age and frailty in a proportional manner, as well as the increase in frailty as the years of education of the elderly decrease. The latter would explain the high levels of frailty among the elderly in Requena, since most of them had only received basic schooling. At the national level, Peru has less frailty (20%) than Requena. In Latin America, the prevalence of frailty syndrome in non-institutionalized elderly people is between 7.7% and 39.3%, which may be associated with different sociodemographic factors [49].

Real et al. [50] emphasized the strong relationship between poor nutritional status, loss of muscle mass, and frailty. Similar results were observed in Requena, where the reciprocity between a poor score on the MNA scale and the development of frailty was found. Another factor associated with frailty is dependence in activities of daily living, that is, presenting a high score on the Barthel scale, coinciding with the results obtained by Herrera-Pérez et al. [49] in Peru.

In a Spanish study [51] it was detected that another possible risk factor for developing frailty was being a woman. In our case, we did not find any relationship between frailty and the sex of the patient. In relation to the protective factors for frailty, it was observed that living in the company of a family member was beneficial for the elderly, which, in Requena, is complicated to apply since the abandonment of the elderly is recurrent.

A good nutritional level is essential to be able to delay the state of dependence as much as possible, since it involves costs at both the health and personal levels [52], not only for the person in this situation, but also for the caregivers who must provide the necessary care. For this reason, it is of great interest to carry out early assessments of all elderly people to establish primary, secondary, and tertiary preventive measures to reduce, as much as possible, the situations that can worsen the quality of life of the elderly.

Among the limitations of this study, we found that, in a considerable percentage of the cases, it was not possible to carry out the assessment of the sphere of mobility and gait alterations due to difficulties in the terrain of the homes of the elderly or due to the physical conditions of the participants, such as blindness and total immobility. Another important limitation is that, given the specificity of the population studied, the generalizability of the findings to other regions or countries could be limited.

## 5. Conclusions

Most of the elderly had a poor nutritional status, a condition related to the deficit in the consumption of highly nutritious foods triggered by the situation of extreme poverty, although their degree of dependence was mostly mild, since a high percentage of the elderly were fully capable of performing all the activities necessary to live in autonomy. The high degree of living alone among the elderly of Requena forced them to maintain their independence. The assessment of their quality of walking was normal, although the conditions of the terrain or housing compromised the safety of the elderly. Nevertheless, the degree of frailty of the elderly in Requena exceeded the national average in Peru, having a negative impact on quality of life and being a predictor of the development of disease. We found a statistically significant association between nutritional status, dependence, and frailty. The better-nourished elderly were less frail and less dependent.

It is necessary to continue assessing the most vulnerable elderly living in impoverished environments and to carry out multidisciplinary approaches. The results obtained will allow personalized recommendations to be made to prevent situations of dependence at the functional level and, clearly, it will be necessary to follow-up with individuals over time to detect and address risk factors to avoid complications. It is of great interest to obtain funding from institutions, universities, companies, and private donations to develop projects focused on improving the nutritional status of the elderly through protein intake, as well as designing a physical exercise plan to improve strength and balance, and prevent falls.

## Figures and Tables

**Table 1:** Sociodemographic characteristics of the sample.

	*n* (%)
Sex	
Male	27 (45.0)
Female	33 (55.0)
District	
Prolongación Requenino	4 (6.67)
Requena	8 (13.34)
Nueva Requena	7 (11.67)
Monopolio	1 (1.67)
Vargas Guerra	4 (6.67)
Jerusalem	4 (6.67)
Aeropuerto	2 (3.34)
San Pedro	2 (3.34)
Jose Carlos Maria Tegui	5 (8.34)
Padre Giner	2 (3.34)
Pedrera	2 (3.34)
Petroperu	3 (5.00)
Emilio San Martin	1 (1.67)
Sancho Roca	3 (5.00)
Union	2 (3.34)
Manaos	1 (1.67)
Sargento Lores	1 (1.67)
Independencia	1 (1.67)
Avenida del ejército	1 (1.67)
Ayacucho	1 (1.67)
San Juan	1 (1.67)
Pedroche	1 (1.67)
Atenas	2 (3.34)
Yanira	1 (1.67)
Allergies	
Yes	3 (5.00)
No	57 (95.0)
Family history	
Yes	16 (26.67)
No	44 (73.33)
Hospitalization in the last five years	
Yes	7 (11.67)
No	53 (88.33)
General physical appearance	
Healthy	25 (41.67)
Dirty–Poor condition	12 (20.00)
Improvable	12 (20.00)
Unkempt	11 (18.33)

**Table 2:** Geriatric assessment test of the sample.

	*n* (%)
Frailty	36 (60.0)
Male	15 (25.0)
Female	21 (35.0)
MNA	
Satisfactory nutritional status	9 (15.0)
At risk of malnutrition	29 (48.3)
Malnutrition	22 (36.7)
Barthel	
Mild dependence	52 (86.7)
Moderate dependence	6 (10.0)
Total dependence	2 (3.3)
Timed get up and go	
Normal	25 (41.7)
Normal for frail people	20 (33.3)
Needed assistance	7 (11.7)
Could not perform	8 (13.3)

**Table 3:** MNA and Barthel scores depending on the state of frailty.

	No (*n* = 24)	Yes (*n* = 36)	*p*-Value *
MNA score (mean (SD))	28.63 (6.05)	17.97 (4.77)	0.019
Barthel score (mean (SD))	89.72 (18.44)	69.79 (11.83)	0.034

* Mann Whitney test.

## Data Availability

Data are available upon reasonable request.
